# Guidance on C-reactive protein point-of-care testing and complementary strategies to improve antibiotic prescribing for adults with lower respiratory tract infections in primary care

**DOI:** 10.3389/fmed.2023.1166742

**Published:** 2023-05-30

**Authors:** Oliver Van Hecke, Lars Bjerrum, Ivan Gentile, Rogier Hopstaken, Hasse Melbye, Andreas Plate, Jan Y. Verbakel, Carl Llor, Annamaria Staiano

**Affiliations:** ^1^Nuffield Department of Primary Care Health Sciences, University of Oxford, Oxford, United Kingdom; ^2^Nuffield Department of Primary Care Health Sciences, NIHR Community Healthcare Medtech and IVD Cooperative, University of Oxford, Oxford, United Kingdom; ^3^Department of Public Health, University of Copenhagen, Copenhagen, Denmark; ^4^Department of Clinical Medicine and Surgery, Section of Infectious Diseases, University of Naples Federico II, Naples, Italy; ^5^Department of General Practice, CAPHRI School for Public Health and Primary Care, Maastricht University Medical Centre, Maastricht, Netherlands; ^6^General Practice Research Unit, Department of Community Medicine, The Arctic University of Norway, Tromso, Norway; ^7^Institute of Primary Care, University of Zurich and University Hospital Zurich, Zurich, Switzerland; ^8^EPI-Centre, Department of Public Health and Primary Care, Academisch Centrum voor Huisartsgeneeskunde, Leuven and NIHR Community Healthcare Medtech and IVD Cooperative, Leuven, Belgium; ^9^Department of Public Health and Primary Care, University of Southern Denmark, Odense, Denmark; ^10^Department of Translational Medical Sciences, University of Naples “Federico II”, Naples, Italy

**Keywords:** CRP, GP, LRTI, antibiotic stewardship, point-of-care test, respiratory tract infections, primary care, antibiotic prescribing

## Abstract

The world faces the threat of increasing antimicrobial resistance, and there is growing consensus that swift action must be taken to improve the rational use of antibiotics and increase the stewardship of antibiotics to safeguard this key resource in modern healthcare. This paper provides the perspective of an international group of experts on the role of C-reactive protein point-of-care testing (CRP POCT) and other complementary strategies to improve antibiotic stewardship in primary care, with regards to the diagnosis and treatment of adult patients presenting symptoms of lower respiratory tract infections (LRTIs). It provides guidance regarding the clinical assessment of symptoms in combination with C-reactive protein (CRP) results, at the point of care, to support the management decision, and discusses enhanced patient communication and delayed prescribing as complementary strategies to decrease the inappropriate use of antibiotics. Recommendation: CRP POCT should be promoted to improve the identification of adults presenting with symptoms of LRTIs in primary care who might gain additional benefit from antibiotic treatment. Appropriateness of antibiotic use can be maximized when CRP POCT is used together with complementary strategies such as enhanced communication skills training and delayed prescribing in addition to routine safety netting.

## 1. Introduction

Antimicrobial resistance (AMR) is broadly recognized as one of the biggest threats to global health, food security, and development today (1–3). AMR develops naturally as bacteria change in response to the use of antibiotics, but overuse and misuse of antibiotics in humans and animals are accelerating the process. Antibiotic-resistant infections have been shown to affect patients’ recovery, symptom severity and indirectly contribute to increasing clinical workload in primary care. A growing number of infections are becoming more difficult to treat as antibiotics become less effective. AMR leads to higher medical costs, prolonged hospital stays, and increased mortality. An estimated 4.95 million deaths have been associated with bacterial AMR in 2019, including 1.27 million deaths directly attributable to bacterial AMR.

Antibiotic stewardship, which implies a more rational prescription and use of antibiotics, is crucial. For humans, the majority of antibiotic prescriptions are issued by primary care physicians (4). A major portion of antibiotic prescriptions issued to adults is for the treatment of respiratory tract infections. A large part of these prescriptions is estimated to be inappropriate, as the majority are self-limiting viral (70%) or bacterial infections (5). C-reactive protein testing (CRP POCT) offers a quick and reliable indicator to better understand the seriousness of an infection, more specifically to understand if an infection is self-limiting or not, and thus to assess if antibiotic treatment would provide any additional benefit to the patient. The use of CRP POCT together with complementary strategies such as enhanced communication skills training and delayed prescribing in addition to routine safety netting, are recommended to maximize the appropriateness of antibiotic use for adults presenting with symptoms of lower respiratory tract infections (LRTI) in primary care.

## 2. C-reactive protein point-of-care testing

### 2.1. General introduction to CRP POCT

The C-reactive protein is a sensitive and non-specific marker for inflammation which can be used to assess the severity of an inflammation and to predict if an infection can be expected to be self-limiting or severe. Self-limiting infections (viral or bacterial) are those that tend to resolve themselves without further treatment and represent the majority of respiratory tract infections seen in primary care. If an inflammation is not severe or appears to be self-limiting - regardless of whether it is caused by a bacteria or virus - antibiotic treatment will not provide additional benefit and may cause undesired side effects and alterations to the microbiome (6). On the other hand, if the severity of respiratory illness is identified as severe, antibiotic treatment can be promptly commenced.

For LRTIs in ambulatory care, the robustness and accuracy of CRP to rule out severe infections are high (7, 8). Specifically in relation to LRTI, the value of adding CRP measurement to a basic signs-and-symptoms prediction model is demonstrated by a metanalysis of over 5,000 adults with suspected LRTI. The accuracy of CRP POCT is comparable to test results obtained using classical laboratory testing (9, 10). While CRP tests can be performed in a centralized laboratory, there are many advantages to having POCT available in the primary care setting. Waiting times for results are drastically reduced or eliminated, allowing clinicians to directly complement their clinical diagnosis with an objective test result. The patient is informed immediately and with convincing arguments, improving the patient experience and adherence to the treatment decision. Furthermore, a POCT can typically be performed by personnel that are not trained in clinical laboratory sciences, such as a nurse or medical assistant (11, 12), if the proper training has been provided.

### 2.2. Evidence that the use of CRP POCT to complement the clinical assessment safely reduces antibiotic prescribing for adults presenting symptoms of LRTIs in primary care

The use of quantitative CRP POCT in the primary care setting to support the decision about antibiotic prescribing for LRTIs has been well evaluated and found to help reduce antibiotic prescribing by up to 42% (relative reduction; 22% absolute reduction, 31% vs. 53%) (13) under trial conditions. Even higher relative reductions of more than 60% of antibiotic prescribing could be achieved when CRP POCT was combined with communication skills training of general practitioners (absolute reduction 44, 23% vs. 67%; RR 0.38; 95% CI 0.36 to 0.55 (14)). Several reviews and meta-analysis, including the Cochrane reviews of Smedemark et al. in 2022 and Tonkin-Crine et al. in 2017, provide further support concluding that CRP POCT reduces the prescribing of antibiotics for acute respiratory tract infections in primary care without compromising patient safety or satisfaction (15–18). The latest Cochrane Review of Smedemark et al. in 2022 covering 11 (cluster-) randomized trials observed a mean reduction of antibiotic prescribing of 24% (RR 0.76; 95% CI 0.68 to 0.86) in adults, Similarly the European Network for Health Technology Assessment found a reduction rate of antibiotic prescribing of 24% (RR 0.76; 95% CI 0.67 to 0.86) for (cluster-) randomized studies and 39% (RR 0.61; 95% CI 0.54 to 0.69) for observational studies. Verbakel et al. calculated for (cluster-) randomized studies a reduction rate of 32% (RR 0.68; 95% CI 0.63 to 0.74) in adults and of 44% (0.56; 95% CI 0.33 to 0.95) in children when cut-off guidance was provided. A 26% relative reduction (20% absolute reduction, 57.0% vs. 77.4%) of antibiotic prescribing rates has also been demonstrated in patients with acutely exacerbated chronic obstructive pulmonary disease (COPD) (19) and a 35% relative reduction (29% absolute reduction, 53.5% vs. 82.3%) has been achieved in older adults in nursing homes (20).

### 2.3. The advantages of using CRP POCT to support clinical assessment, diagnosis, patient management and treatment decision processes

CRP POCT adds to the complete understanding of a patient’s situation and reduces subjectivity when assessing the severity of an infection and deciding on further patient treatment or management. CRP values should always be interpreted together with a thorough assessment of the patient’s history, risk-profile, and acute clinical situation.

A clinician (doctor or nurse) may use a CRP POC test to help assess the severity of a potential LRTI and reduce diagnostic uncertainty as part of their clinical assessment. For example, CRP POCT can be useful in LRTIs where there is clinical uncertainty as to whether there is a serious LRTI (21, 22).

The results of a CRP POC test can be relevant in the majority of adult patients presenting with symptoms of LRTIs in primary care. When the prevailing consideration is to prescribe antibiotics, a CRP POC test can give a physician more certainty and avoid an antibiotic prescription motivated by a ‘better safe than sorry’ or ‘just-in-case’ approach. And similarly, when the prevailing consideration is not to prescribe antibiotics, a CRP POC test can be used to help rule out a potentially serious LRTI.

Additionally, the CRP results can be used for explanation and support patient-doctor communication, especially when a patient asks for antibiotics. This reinforcement of patient communication is particularly important in regions with relatively high antibiotic use, and easy (over-the-counter) access to antibiotics.

While the use of CRP POCT is recommended, the decision whether to perform a CRP POC test and whether to prescribe antibiotics, is and should always remain at the discretion of the physician depending on patient characteristics and available resources.

### 2.4. Considerations of certainty

There are cases when a physician may feel that a CRP POC test is clearly not needed, e.g., common cold. However, the idea of limiting the use of CRP POCT to cases when a physician is uncertain about their treatment decision may leave the potential benefits of CRP POCT underutilized to optimize antibiotic prescribing. The clinical diagnosis of pneumonia in general practice is often incorrect (23). Moreover, physicians are generally overconfident in their antibiotic prescribing decision. During an audit of 4,982 consultations with sore throat and/or LRTIs across 18 countries, GPs rated their level of confidence as certain or very certain in 90% of consultations; however, prescribing rates among these clinicians were higher than what is considered appropriate (24). Other research has shown that 46% of the antibiotics prescribed for adults with LRTIs were not indicated by guidelines (over prescription) (25). This further underlines the potential disconnect between confidence levels and appropriateness. It is for this reason that it is recommended to perform a CRP POC test to confirm the antibiotic prescribing decision in LRTIs. Confirmation or re-evaluation of a treatment decision is a learning opportunity and will contribute over time to better antibiotic prescribing behavior (26) and overall antibiotic stewardship.

### 2.5. Interpretation of CRP results

The figure below represents CRP value thresholds and the corresponding recommended prescribing considerations, in line with several national guidelines (27–42) and various meta-analyzes and systematic reviews. While these ranges are broadly applicable, the importance of an individual evaluation of each patient is key and should lead the final treatment decision. Patient specifics such as relevant comorbidities (i.e.: COPD, diabetes, etc.) or other sources of vulnerability and risk should be considered.

The following treatment considerations are recommended for the CRP ranges listed below, when treating adult patients presenting in primary care with symptoms of LRTIs:

• < 20 mg/L (~74% of patients (43)): It is strongly recommended not to prescribe antibiotics
• For the ‘grey zone’ ranging from 20 to ≤ 100 mg/L (~23% of patients), the clinical picture is most deciding for any treatment considerations. In most cases, antibiotics are not needed. Prescribing antibiotics should be considered in patients with relevant comorbidities, such as COPD, diabetes and in vulnerable elderly, and re-consultation or delayed prescribing should be considered when relevant.

o 20 to ≤ 40 mg/L: It is generally recommended not to prescribe antibiotics

▪ Exception: cases where patients are exhibiting COPD exacerbation with obvious increased purulence of sputum or for patients that are at high risk of deterioration due to other relevant comorbidities

o 40 to ≤ 100 mg/L: The clinical picture is most deciding for any treatment considerations. In most cases, antibiotics are not needed when there are no relevant comorbidities. Antibiotics may be considered in cases where a severe (non-self-limiting) bacterial infection is suspected, cases where patients are exhibiting COPD exacerbation with obvious increased purulence of sputum or for patients that are at high risk of deterioration due to other relevant comorbidities.

• >100 mg/L (~3% of patients): It is strongly recommended to start treatment with antibiotics, due to a high risk of a severe bacterial infection. Consider hospital referral according to clinical evaluation.

### 2.6. Interpretation of CRP results during active pandemics/epidemics

First evidence indicates that CRP values are mostly elevated in COVID-19 infections. In cases of COVID-19 infections, antibiotics are not recommended (44, 45). For patients that have tested positive for COVID, CRP POCT can be useful for disease prognosis, as research has shown that raised CRP >40 mg/L is indicative of severe infections and complicated courses, indicating a need for close follow-up or hospitalization (46, 47).

In the presence of cough and fever during an active pandemic or epidemic (such as COVID-19 or Influenza A&B), it may be useful to rule out the disease in question with a rapid viral test when available before further interpretation of CRP results.

## 3. Communication strategies to increase antibiotic stewardship

A CRP test-result and a well-informed prescribing decision the first steps toward reducing antibiotic over-consumption, but patients need to be informed, and play a key role in antibiotic stewardship. The physician-patient consultation is arguably one of the most important communication moments, due to its acute relevance, making the interacting parties more open to receive relevant messages. Below are some strategies to strengthen the desired effect of communications.

### 3.1. Review decision aids together with the patient

Reviewing a decision aid together with a patient can be very beneficial as it improves patient knowledge and understanding of decisions and increases engagement by enforcing the feeling of a shared decision-making process. Research has shown that using decision aids do not result in the worsening of patient satisfaction or health outcomes and have the potential to contribute to further reductions in antibiotic prescribing (up to 9,1%) (48). Research has shown that the review of a decision aid adds on average 2.6 min to a standard consultation time (49), but it may be expected to relieve pressure on the practice over the long term, as patients learn not to consult with their GPs too quickly after the onset of symptoms.

### 3.2. Use the announcement method

Research has shown that the words of a treating physician carry significant weight in influencing patient behavior (50). The announcement method simply states that physicians should give a clear and strong message to the patients, recommending a course of action. In cases where the clinical evaluation of a physician leads to the decision not to prescribe antibiotic treatment, simple and clear statements like the following could be impactful.

- “For your current situation, you should not take antibiotics.”
- “We should not start an antibiotic treatment; it could do more harm than good.”

Here below are selected messages that could be beneficial to give during the physician-patient interaction. These messages could be given at different moments of a consultation, and do not represent a monolog, but rather talking points to integrate when relevant and in the natural flow of a physician-patient conversation. Naturally, these messages should be preceded by an exploration of the patient’s symptoms, ideas and concerns, and a clinical examination. It is also useful to discuss the patient’s beliefs, attitudes, or expectations with respect to antibiotic treatment.

**Table 1:** 

Main messages to be delivered by the physician to the patient	Explanation and additional details
Our clinical assessment of your situation could benefit from a CRP test; if the value is low, it means that you have a mild infection (often viral).	Discuss leaflet or decision aid, and state that if
	• CRP is low, it indicates a minor infection (often viral) and no antibiotics are needed.
	• Consider delayed prescribing where applicable (ie: in the grey zone from Figure 1)
Good news! Your CRP is low. …So you should not take antibiotics. *(announcement method)*	Practice safety-netting: if symptoms get worse or the condition changes, or if in doubt, re-consultation may be advised
How do we know when we need to use antibiotics? The CRP value tells me if your inflammation is so severe, that you need an antibiotic today to help your body to fight.	• Discuss CRP cut-off values, then explain:
	• The presence of cough or fever does not necessarily mean that you need antibiotics.
	• Antibiotics do not work for viruses.
	• Antibiotics may do more harm than good when an infection is non-severe.
It is not abnormal for a cough to persist for quite a long time (up to 6 or even 8 weeks), and antibiotics will not help to shorten this period.	• A cough of 5–7 days is not at all abnormal or necessarily alarming.
	• Average duration of a cough is 3 weeks, but is often up to 5 weeks; even up to 8 weeks is not necessarily alarming.

**Figure 1:**
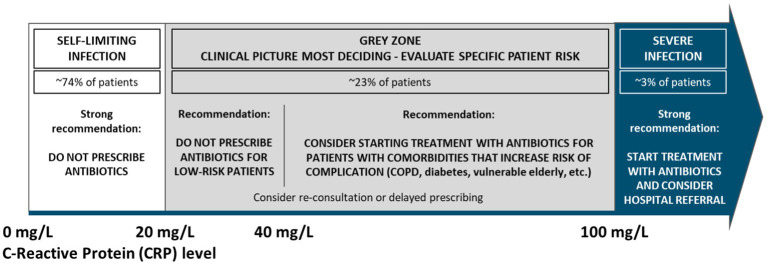
CRP thresholds for adults presenting symptoms of LRTIs.

### 3.3. Training in communication skills

While the use of CRP POCT reduces antibiotic prescribing by up to 42% (relative reduction; 22% absolute reduction, 31% vs. 53%)additional studies in primary care have demonstrated that combining CRP POCT with communication skills training can significantly increase this impact. Recommended focus points for communication skills training are patient-centered consultation, and shared decision-making (physician-patient) techniques.

Patient-centered consultation aims for a more individualized approach to patient care, that emphasizes respect for individual preferences and patient empowerment. It addresses topics such as how to ask for information about patient conditions, symptoms, and concerns and how to listen actively, openly, and in an unbiased way to the patient.

Shared decision-making techniques (physician-patient) aim to involve the patient more actively in the decision-making process, leading to a mutual decision in the best interest of the patient. The decision-making process combines evidence-based information with the clinical evaluation and experience of the physician, but also the patient’s culture, values, and individual preferences.

## 4. Delayed prescription

Delayed prescribing is another tool at the disposition of a clinician that can contribute to better antibiotic stewardship and the reduction of antibiotic over-use in a safe way, when the circumstances and patient specifics warrant it. Delayed prescribing is when the patient receives an antibiotic prescription with the instruction to “delay fulfilling it” for a certain amount of time, agreed upon between the physician and the patient (typically 2–3 days but up to 7 days for LRTIs), during which symptomatic treatment may be started. If symptoms persist or get worse during that period, the patient can fulfill the antibiotics prescription at their own discretion or re-evaluate with the physician.

Delayed prescribing can be considered when patients visit the physician after 3–5 days of symptoms and have a CRP value between 20 and 100 mg/L (the ‘grey zone’). Additionally, the clincians’s assessment should conclude that the patient is not at higher d risk of deterioration (i.e., due to existing conditions or severe symptoms) and can be relied upon to follow the prescription fulfillment instructions.

Delayed antibiotic prescribing is considered a safe and effective strategy.

• An individual patient data meta-analysis demonstrated a 16% re-consultation rate when delayed prescribing was done, while immediate prescribing resulted in a 22% re-consultation rate and no significant difference was found in the severity of the symptoms between delayed and immediate antibiotics prescribing two to 4 days after the consultation (51).
• A 2017 Cochrane review demonstrates that delayed prescription of antibiotics for RTIs has resulted in significantly lower rates of antibiotic use compared to cases of immediate antibiotics prescription (31% versus 93%) (52).
• A randomized controlled trial with 258 patients (107 LRTIs and 151 rhinosinusitis) carried out by 32 family physicians in the Netherlands combined CRP POCT and delayed prescribing. Delayed prescriptions given to patients based on CRP assistance resulted in an absolute 49% lower fill rate compared with delayed prescription in the control group (53).
• A recent observational study on delayed prescribing carried out in three primary care centers in Spain also resulted in a reduction in antibiotic use for acute bronchitis and acute pharyngitis compared to immediate antibiotic prescription. Despite not asking about the actual consumption of antibiotics, 64.3% of the patients filled the delayed prescription at the pharmacy or declared taking another antibiotic (54). The reduction in antibiotic prescribing was lower than in randomized clinical trials, being comparable to the results obtained with other observational studies on delayed antibiotic prescribing. In addition, only a few patients adhered to the doctors’ instructions. Observational studies may better reflect daily practice.

A thorough risk assessment including both individual and systemic factors is key when deciding whether to use a delayed prescribing approach.

## 5. Discussion

AMR is a global threat that must urgently be addressed. Using CRP POCT to better discriminate between severe and non-severe infections, complemented with effective patient communication and when applicable delayed prescribing, are part of the solution to improve antibiotic stewardship in primary care. There is a clear added value to embedding CRP POCT in the routine clinical decision-making process for adults with LRTIs in primary care. That said, it should be noted that CRP is always a compliment to, and cannot replace, a thorough clinical assessment.

While current evidence highlights the opportunity to improve antibiotic stewardship, broader adoption of CRP POCT in the routine primary care remains limited to a few European countries. To help bridge the gap between evidence and practice urgent actions are needed with all relevant stakeholders to establish high-quality CRP POCT in routine practice. Context-specific research may be needed to demonstrate feasibility and (cost)effectiveness in specific settings.

Successful implementation of CRP POCT in general practice will ask for significant changes in professional behavior. Behavioral change will require the trust of GPs in the new diagnostic tool. It is therefore important that introduction is guided with proper instruction and training of the users and evaluation of the first experiences of GPs, nurses and patients. Sufficient reimbursement and guidelines for use may not be enough to encourage a broad and effective adoption of CRP POCT. Supporting policy and frameworks from regulators would be helpful to accelerate implementation. CRP POCT can only be reliable and safe, when accompanied with operating procedures and quality-assurance monitoring, according to (inter)national norms and standards. Although testing devices can be very reliable, performance is mainly compromised by human errors and logistic issues throughout the full process of high-quality point-of-care testing. Collaborations between POCT experts and primary care professionals, ideally including automated communication processes to check for and act on possible errors made by the users of CRP POC tests, would be beneficial. An effective implementation of CRP POCT may require country-specific organization and implementation strategies. Important stakeholders of a country’s healthcare ecosystem, including local and national authorities, should look for ways to facilitate sustainable implementation, so that AMR can be combatted effectively.

## 6. Conclusion

Using CRP POCT, when applied together with clear guidance, advanced communication strategies and safety netting techniques, and delayed prescribing, can significantly improve the rational prescription and use of antibiotics for adults presenting in primary care with symptoms of LRTIs.

In addition to a thorough clinical assessment of the patient, the evaluation of CRP can help primary care clinicians understand if infections are self-limiting or severe, and thus evaluate the usefulness of antibiotics prescribing for LRTIs. CRP POCT has been proven to significantly reduce antibiotic prescribing and is therefore recommended by several national guidelines. A broader adoptation of these technologies and techniques is strongly recommended.

## Data availability statement

The original contributions presented in the study are included in the article/supplementary material, further inquiries can be directed to the corresponding author.

## Author contributions

All authors listed have made a substantial, direct, and intellectual contribution to the work and approved it for publication.

## Funding

The work was funded by Abbott Rapid Diagnostics. The funder was not involved in the study design, collection, analysis, interpretation of data, the writing of this article, or the decision to submit it for publication.

## Conflict of interest

Outside the scope of this article, IG has acted as a consultant for MSD, Gilead, Abbvie, Pfizer, Angelini, Nordic, GSK, and SOBI. RH declares to have received honoraria from Abbott, LumiraDx, Future Diagnostics, Photondelta, Cepheid and Roche for advisory board meetings and lecturing. Outside the scope of this article, AS has acted as a consultant for Aboca, Angelini and Novalac; as clinical investigator for Janssen Biologics B.V. and Novalac; speaker for Novartis, Bromatech, Sanofi, Vyvalife; and was clinical investigator for Aboca, Eli Lilly Cork Limited and PAREXEL International Srl. CL declares to have received honoraria from Abbott for lecturing.

The remaining authors declare that the research was conducted in the absence of any commercial or financial relationships that could be construed as a potential conflict of interest.

## Publisher’s note

All claims expressed in this article are solely those of the authors and do not necessarily represent those of their affiliated organizations, or those of the publisher, the editors and the reviewers. Any product that may be evaluated in this article, or claim that may be made by its manufacturer, is not guaranteed or endorsed by the publisher.

## Abbreviations

AMR: Antimicrobial Resistance
CRP: C-Reactive Protein
GP: General Practitioner
LRTI: Lower Respiratory Tract Infection
POCT: Point-of-Care Testing
RTI: Respiratory Tract Infection
RR: Risk Ratio
